# Host Resistance to Bacterial Infection Varies Over Time, but Is Not Affected by a Previous Exposure to the Same Pathogen

**DOI:** 10.3389/fphys.2022.860875

**Published:** 2022-03-21

**Authors:** Beatriz Acuña Hidalgo, Sophie A. O. Armitage

**Affiliations:** Institute of Biology, Freie Universität Berlin, Berlin, Germany

**Keywords:** bacterial pathogen, *Drosophila melanogaster*, heat-killed bacteria, formaldehyde inactivated bacteria, immune priming, resistance, survival, innate immunity

## Abstract

Immune priming describes the phenomenon whereby after a primary pathogen exposure, a host more effectively fights a lethal secondary exposure (challenge) to the same pathogen. Conflicting evidence exists for immune priming in invertebrates, potentially due to heterogeneity across studies in the pathogen species tested, the antigen preparation for the primary exposure, and the phenotypic trait used to test for priming. To explore these factors, we injected *Drosophila melanogaster* with one of two bacterial species, *Lactococcus lactis* or *Providencia burhodogranariea*, which had either been heat-killed or inactivated with formaldehyde, or we injected a 1:1 mixture of the two inactivation methods. Survival and resistance (the inverse of bacterial load) were assessed after a live bacterial challenge. In contrast to our predictions, none of the primary exposure treatments provided a survival benefit after challenge compared to the controls. Resistance in the acute phase, i.e., 1 day post-challenge, separated into a lower- and higher-load group, however, neither group varied according to the primary exposure. In the chronic phase, i.e., 7 days post-challenge, resistance did not separate into two groups, and it was also unaffected by the primary exposure. Our multi-angled study supports the view that immune priming may require specific circumstances to occur, rather than it being a ubiquitous aspect of insect immunity.

## Introduction

Research on invertebrate immune defenses over the past few decades has changed our understanding of immune memory. The definition of immune memory has been extended beyond a phenomenon restricted to vertebrate adaptive immunity to include invertebrates, plants and bacteria (Pradeu and Du Pasquier, 2018). In the case of invertebrates, evidence for a memory-like phenomenon has been found across a broad range of taxa (Contreras-Garduno et al., 2016; Milutinović and Kurtz, 2016; Pradeu and Du Pasquier, 2018). This phenomenon, termed “immune priming” (Little and Kraaijeveld, 2004), has been described as the ability of an immune system to store or use the information on a previously encountered antigen or parasite, upon a secondary exposure (Kurtz, 2005; Milutinović and Kurtz, 2016).

There is considerable evidence supporting immune priming in invertebrates [reviewed in Contreras-Garduno et al. (2016) and Milutinović and Kurtz (2016)], with one mechanistic basis being haemocyte-mediated defences (Pham et al., 2007; Rodrigues et al., 2010). However, a number of studies testing immune priming have not found evidence to support its existence (Pham et al., 2007; Reber and Chapuisat, 2012; Longdon et al., 2013; Wu et al., 2015; Duneau et al., 2016; Patrnogic et al., 2018; Kutzer et al., 2019) [reviewed in Contreras-Garduno et al. (2016) and Milutinović and Kurtz (2016)]. It has been suggested that the inconsistent findings are due to heterogeneity in the way in which this phenomenon has been tested across studies (Milutinović and Kurtz, 2016; Pradeu and Du Pasquier, 2018). Although the following list is by no means exhaustive, heterogeneity has come in the form of variation in the pathogen species tested, the methods used to prepare the pathogen for the previous exposure, and the phenotypic read-out used to assess whether there is evidence for priming or not. Our experimental design encompasses testing variation in all three of these factors.

First, evidence that priming could be pathogen species dependent comes from studies where within one experiment, priming has been found against one species of pathogen but not against another, for example Pham et al. (2007) and Roth et al. (2009). The evolutionary history and ecology of the host-pathogen interaction studied might also play a role. For example, previous exposure to a gram-positive bacterium conferred *Tenebrio molitor* a more effective protection against infection compared to a gram-negative bacterium (Dhinaut et al., 2018). The authors suggested that since many pathogenic bacteria naturally present in the environment of *T. molitor* are gram-positive, immune priming might have only evolved against these bacteria as they represent a significant threat to the host (Dubuffet et al., 2015; Dhinaut et al., 2018). Here we test two species of bacteria, both isolated from the host species.

Second, Milutinović and Kurtz (2016) proposed that using different antigen preparation methods for the primary exposure might result in the antigens being recognised in contrasting ways by the host immune system, leading to inconsistent results between studies. Antigen preparations have ranged from cell components and toxins (Karp and Rheins, 1980; Rheins et al., 1980; Milutinović et al., 2014; Miyashita et al., 2014, 2015) to varying doses of live (Castro-Vargas et al., 2017; Chambers et al., 2019) or inactivated pathogens (Pham et al., 2007; Lin et al., 2013). The use of live compared to dead pathogens for the primary exposure might lead to different priming responses (Milutinović and Kurtz, 2016). A live primary infection can lead to an initial phase of host mortality, after which survivors are challenged with a secondary infection. This first exposure may act as a filter, selecting for fitter hosts. Compared to non-primed individuals, these hosts are predicted to survive the challenge better due to their higher fitness, rather than an ability to store and recall information on a previous encounter with the pathogen (Kurtz, 2005; Milutinović and Kurtz, 2016). Moreover, a live pre-exposure could lead to a persistent infection and result in differential bacterial loads across hosts (Acuña Hidalgo et al., 2021) and thereby introduce heterogeneity in the immunological history of the pre-exposed flies. Therefore, in common with the majority of studies examining immune priming. We here focus on the use of inactivated pathogens.

Pathogens can be inactivated using a number of methods including heat-killing (González-Tokman et al., 2010; Wu et al., 2014; Riessberger-Gallé et al., 2015; Kutzer et al., 2019) and chemical compounds like formaldehyde (Wang et al., 2009; Zhuang et al., 2011; Dhinaut et al., 2018) and glutaraldehyde (Faulhaber and Karp, 1992; Rosengaus et al., 1999). There are a limited number of studies directly comparing whether the antigenic preparation method affects the likelihood of uncovering a priming effect (Lin et al., 2013; Miyashita et al., 2014). Lin et al. (2013) found that the immune system of the white shrimp *Litopenaus vannamei* is activated more quickly by heat-killed *Vibrio alginotylicus*, but that the response induced after challenge is stronger and induces a higher resistance to live bacteria in shrimp primed with formalin-inactivated *V. alginotylicus.* The authors argued that this might be due to how the inactivation methods affect the antigenicity of the bacterial cells (Lin et al., 2013). Heating bacterial cells can lead to membrane disruption (Russell, 2003), releasing lipopolysaccharides (Katsui et al., 1982; Tsuchido et al., 1985), which may act as immunostimulants for the host (Lin et al., 2013). This would lead to a fast response but cause the bacterial cells to retain less antigenicity (Lin et al., 2013). On the other hand, formaldehyde cross-links the molecules present on the surface of the cell (Fraenkel-Conrat and Olcott, 1948; Feldman, 1973) leading to formalin-inactivated bacteria to retain a high level of antigenicity (Spitznagel and Trainer, 1949; Arshadi et al., 2020).

Third, the phenotypic trait that has been measured to test whether there is increased protection upon the secondary encounter, varies across studies. This protection has most frequently been tested by monitoring survival after the secondary exposure (Contreras-Garduno et al., 2016), showing an increased longevity in some (Boman et al., 1972; Faulhaber and Karp, 1992; Pham et al., 2007; Roth et al., 2009; Christofi and Apidianakis, 2013; Lin et al., 2013; Miyashita et al., 2014; Wu et al., 2014; Futo et al., 2016; Castro-Vargas et al., 2017; Lafont et al., 2017; Dhinaut et al., 2018), but not all previously exposed hosts (Boman et al., 1972; Pham et al., 2007; Kutzer et al., 2019). In the traditional sense of immune memory, it would be expected that this increased survival results from the host immune system inducing a stronger and more efficient immune response upon secondary exposure (Pradeu and Du Pasquier, 2018), which can be quantified at the level of the host immune effectors (Lin et al., 2013; Zhao et al., 2013; Wu et al., 2014; Vargas et al., 2020). These changes in the immune response are expected to increased host resistance to the infection, which is defined as the host ability to reduce the pathogen load (Råberg et al., 2009; Graham et al., 2011). Increased resistance upon secondary exposure has been demonstrated (Boman et al., 1972; Sadd and Schmid-Hempel, 2006; Pham et al., 2007; Miyashita et al., 2014), but a primary exposure can also lead to a reduction in host resistance (Kutzer et al., 2019), potentially because a host can tolerate an infection instead of eliminating it (Kutzer and Armitage, 2016b). Despite its relevance as a phenotypic read-out for immune priming, host resistance has not frequently been assayed. Furthermore, while chronic infections can persist in insects for weeks (Haine et al., 2008; Hotson and Schneider, 2015; Acuña Hidalgo et al., 2021), the effects of a primary exposure on resistance post-secondary exposure are not well-understood in the chronic infection phase (Vargas et al., 2020; but see Rodrigues et al., 2010; Contreras-Garduño et al., 2015; Kutzer et al., 2019), with some studies showing that pathogens are not always eliminated in primed hosts (Rodrigues et al., 2010; Contreras-Garduño et al., 2015; Kutzer et al., 2019).

Here, using *D. melanogaster* as our host, we explored the effect of pre-exposure to two bacterial species isolated from wild flies, gram-positive *L. lactis* (Lazzaro, 2002) and gram-negative *P. burhodogranariea* (Juneja and Lazzaro, 2009), which are considered opportunistic pathogens and are able to establish an infection in the fly with lethal consequences for a proportion of the infected flies (Lazzaro, 2002; Lazzaro et al., 2006; Galac and Lazzaro, 2011; Kutzer and Armitage, 2016a; Acuña Hidalgo et al., 2021). We asked whether pre-exposure affords protection against each species of bacteria, and whether the inactivation method affects the level of protection. We hypothesised that flies simultaneously pre-exposed to formaldehyde-inactivated and heat-killed bacteria would benefit from both types of antigenicity and show a higher level of protection compared to a pre-exposure with only one method of inactivation. We also asked whether pre-exposure affects survival and resistance after a homologous challenge with live bacteria. By quantifying resistance as the pathogen load in the acute and chronic phases of infection (1- and 7-days post-infection), we aimed to determine the strength and duration of the immune priming response, as well as its effect on bacterial persistence (Pradeu and Du Pasquier, 2018).

## Materials and Methods

### Experimental Animals

We used an outbred population of *D. melanogaster*, naturally infected with the intracellular bacterium *Wolbachia* (gift from Élio Sucena). This population was established from 160 fertilised females collected in Azeitão, Portugal in 2007 (Martins et al., 2013). The flies were reared and maintained at a density of at least 5,000 flies inside a population cage with non-overlapping generations of 14 days on a 12:12 h light-dark cycle, at 60–80% relative humidity and a temperature of 24.8 ± 0.5°C. They were maintained on a sugar yeast agar medium [SYA medium: 970 mL water, 100 g brewer’s yeast, 50 g sugar, 15 g agar-agar, 30 mL 10% Nipagin solution and 3 mL propionic acid (Bass et al., 2007)].

Experimental flies were produced after two generations of density control. The first density-controlled generation was obtained by placing four grape juice agar plates [25 g agar-agar, 300 mL red grape juice, 21 mL 10% Nipagin solution, 550 mL water (Wensing et al., 2017)] coated with a thin layer of baker’s yeast paste, inside the population cage and letting the flies lay eggs for 24 h. Larvae were collected 24 h after the end of the oviposition period and placed in groups of 100 larvae in plastic vials (95 mm × 25 mm) containing 7 mL of SYA medium. They were left to develop for 8 days under standard conditions. The second density-controlled generation was produced by placing 4-day old adults in two embryo cages, allowing 600–800 adults per cage to oviposit on a grape juice agar plate for 24 h. Larvae were again collected 24 h later at a density of 100 larvae per vial and allowed to develop. One day after the start of eclosion, adults were collected, placed in vials in mixed sex groups of five males and five females.

### Preparation of the Bacterial Solutions

In this study, we used two bacterial species isolated from wild-caught *D. melanogaster* (gifts from Brian Lazzaro), *L. lactis* (Lazzaro, 2002), and *P. burhodogranaria* strain B (Juneja and Lazzaro, 2009) (DSMZ; type strain: DSM-19968). Culturing of these bacteria was performed as in Kutzer and Armitage (Kutzer and Armitage, 2016a). In brief, bacteria were streaked on lysogeny broth (LB) agar directly from aliquots stored in 34.4% glycerol at −80^°^C. After an incubation period of 24 h at 30^°^C, four colony-forming units (CFUs) were added to 100 mL of sterile LB medium and incubated at 30^°^C and 200 rpm. Two individual bacterial cultures were incubated per bacteria. The next morning, approximately 15 h later, the liquid cultures were prepared for the primary exposure or challenge injections.

#### Preparation of Inactivated Bacteria for Primary Injections

After the incubation period of 15 h, the bacteria were centrifuged at 21^°^C and 2,880 rcf for 5 min and washed two times in *Drosophila* Ringer’s solution [182 mmol L^–1^ KCl; 46 mol L^–1^ NaCl; 3 mmol L^–1^ CaCl_2_; 10 mmol L^–1^ Tris⋅HCl (Werner et al., 2000)]. The optical density (OD) of 500 μL of each species was measured using an Ultrospec10 classic spectrophotometer (Amersham, 600 nm), and the OD values were averaged to calculate the total bacterial concentration of the overnight cultures. For each bacterial species we had pre-determined the relationship between OD and the number of live bacteria by plating serial dilutions of bacterial solutions with known ODs. The bacteria were centrifuged again at 2,880 rcf and 21^°^C for 10 min. The supernatant was discarded, and the remaining pellet was resuspended in sterile distilled water. No washing steps with distilled water were performed to limit the exposure of bacteria to osmotic lysis.

For formaldehyde inactivation, a solution containing 5% formaldehyde in sterile distilled water was added to the bacterial solution to achieve a final concentration of 0.5% formaldehyde (Dhinaut et al., 2018). The solution was then placed on a shaker (Biosan ES20) at 1,000 rpm at room temperature. We previously determined the inactivation time needed for each of the two bacterial species by exposing an overnight culture to 0.5% formaldehyde for 10, 30, 120 min or 24 h at room temperature, then plating the bacterial solutions on LB agar plates in triplicate for each time tested, and then verifying the absence of colonies after 24 and 48 h. Formaldehyde inactivates the bacterial cells by cross-linking proteins of the cell wall (Fraenkel-Conrat and Olcott, 1948; Feldman, 1973). Our aim was to kill the cells while preserving the conformation of the membrane as much as possible, and thus the antigenicity of the bacteria, therefore we aimed for the shortest amount of time possible. No colonies grew on the agar plates after 2 h of exposure to formaldehyde for *L. lactis*, and after 10 min of exposure for *P. burhodogranariea*. The inactivated bacterial solution was centrifuged at 21^°^C, at 2,880 rpm for 10 min, the supernatant was removed and the pellet was resuspended in 7 ml Ringer’s solution. This step was repeated two more times to remove the formaldehyde from the solution. To verify that the bacteria had been inactivated, we plated 100 μL of the solution onto LB agar plates and checked for the absence of bacterial colony growth after an incubation period of 24 and 48 h at 30^°^C. The solution was then aliquoted into 1.5 mL microcentrifuge tubes, snap frozen in liquid nitrogen and stored at −80^°^C. Since the bacterial solution was washed in Ringer’s solution three times, we expected that a portion of the inactivated bacterial cells might have been lost during that process. Hence, we once again measured the concentration of the solutions. One tube per bacteria was defrosted at room temperature and serially diluted in *Drosophila* Ringer’s, and the cells were counted using a haemocytometer (Thoma, 0.02 mm deep, 0.0025 mm^2^).

For heat-killing, the bacterial solution was serially diluted to achieve double of the aimed concentration, i.e., 2 × 10^8^ CFUs/mL. The solution was pipetted into several 1.5 mL microcentrifuge tubes and placed on a heat-block (Eppendorf ThermoMixer^®^ C) at 90^°^C and 1,000 rpm. Prolonged exposure to heat can lead to protein denaturation, and thus reduce recognition of the antigens present in the solution by the host immune system. Therefore, we tested for the shortest amount of heat-killing time that would lead to the inactivation of the bacterial cells. The time needed to kill each of the two bacteria was previously tested by exposing them to this treatment for 5, 10, and 20 min. The bacterial solutions were then plated in triplicates on LB agar plates to verify the absence of colonies after 24 h, and then again after 48 h. No *L. lactis* colonies grew on the plates after 10 min of heating, and no *P. burhodogranariea* colonies grew after 5 min of heating. The bacterial solutions were then aliquoted into 1.5 mL microcentrifuge tubes. The final solutions for the injections were made by adding double concentrated Ringer’s solution to the tubes in a 1:1 volume ratio, diluting the concentration to 1 × 10^8^ CFUs/mL. Subsequently, 100 μL per tube were plated onto LB agar and checked for the absence of bacterial colony growth after 24 h of incubation at 30^°^C. The tubes containing the inactivated bacteria were frozen in aliquots in liquid nitrogen and stored at −80 C until use.

Before injection of the primary exposure, the inactivated bacterial aliquots were allowed to defrost at room temperature. For the formaldehyde-inactivated bacteria, three serial dilutions per bacteria were performed and pooled together to adjust the solution to a concentration of 1 × 10^8^ CFUs/mL. For the combination treatment, an equal volume of the formaldehyde-inactivated and heat-killed bacteria were pooled together. A volume of 50 μL of each solution was plated onto LB agar to confirm the absence of bacterial colony growth.

#### Bacterial Preparation for Challenge Injections

The experiment was performed in five independent experimental replicates, and for each experimental replicate, the overnight bacterial cultures were produced following the same protocol as described above. After an incubation period of 15 h, the liquid cultures were centrifuged at 2,880 rcf and 4^°^C for 10 min. The supernatant was removed, and the cultures were washed twice with *Drosophila* Ringer’s solution. The concentration of the bacterial solution was estimated measuring the optical density of 500 μL of the bacterial solution after serial dilution. We aimed to infect the flies with a dose that caused an intermediate mortality, i.e., 50–60% of dead flies by day seven, therefore the concentration was adjusted to 5 × 10^6^ CFUs/mL for *L. lactis* and to 5 × 10^7^ CFUs/mL for *P. burhodogranariea* (Acuña Hidalgo et al., 2021). To verify these concentrations, we performed three serial dilutions of the bacterial solution from 1:1 to 1:10^4^, plated 5 μL of the solution onto agar eight times and counted the number of CFUs that grew after incubation for 20 h at 30°C.

### Previous Exposure and Challenge Injections

**FIGURE 1:**
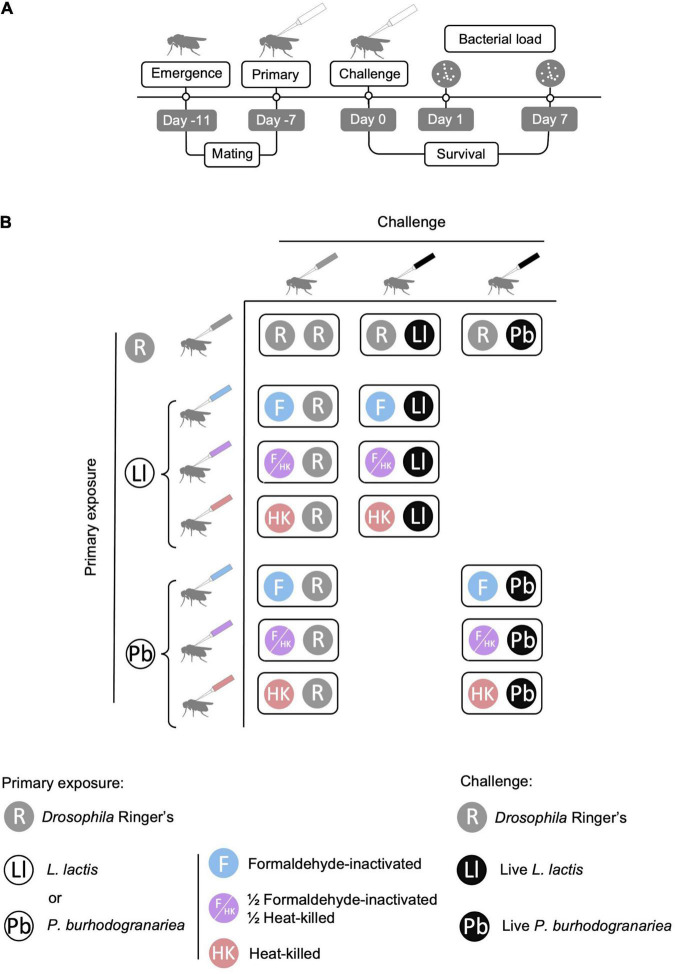
Experimental design. **(A)** Timeline of the experiment with essential steps and assaying timepoints. Emergence refers to the time at which the adults eclosed. **(B)** Previous exposure and challenge treatment combinations used in the experiment. The primary exposure was either to *Drosophila* Ringer’s solution (R), *Lactococcus lactis* (Ll) or *Providencia burhodogranariea* (Pb). The primary bacterial exposure was either formaldehyde inactivated (F), a mixture of formaldehyde-inactivated and heat-killed bacteria (F/HK), or heat-killed bacteria (HK). For each primary exposure-challenge combination treatment flies were challenged with live bacteria; either 92 colony forming units (CFUs) of *L. lactis* or 920 CFUs of *P. burhodogranariea*. Figure adapted from Kutzer et al. (2019).

Of the five independent experimental replicates, three replicates assessed the effect of pre-exposure on survival and bacterial load, and two replicates assessed only the effect on survival. Four days after having been placed in vials with five males and five females, females were exposed to a pre-exposure injection, and then to a challenge injection after 7 days (Figure 1). The previous exposure injections were performed in a randomised block design. Flies were anesthetised with CO_2_ for a maximum of 5 min in groups of 10 flies. A total volume of 18.4 nL of the primary exposure solution containing 1 × 10^8^ CFUs/mL [resulting in around ∼1,840 CFUs injected per fly (Kutzer et al., 2019)] was injected on the right side of the thorax using a fine glass capillary (Ø 0.5 mm, Drummond), pulled to a fine tip with a Narishige PC-10, and connected to a Nanoject II™ injector (Drummond). Flies were injected with one of the three previous exposure treatments per bacteria, i.e., formaldehyde-inactivated bacteria (F), heat-killed bacteria (HK) or a solution containing equal volumes of the two types of inactivated bacterial solutions (F + HK). Control flies were injected with 18.4 nL of *Drosophila* Ringer’s solution (R). In total, per each pre-exposure treatment with dead bacteria, 260 flies were injected (40-60 flies per experimental repeat), and 460 flies were given a control injection with Ringer’s (80–100 flies per repeat). Flies were then transferred to vials containing 7 mL of fresh SYA medium, kept in groups of 10 at 25^°^C and 70% relative humidity and flipped into new food vials every 2–4 days. For each group of 10 flies, one aliquot containing the injection solution was used. At the end of the injections, the remaining volume of each aliquot was plated onto LB agar and incubated at 30^°^C for 15 h to confirm that there was no contamination. No CFUs grew on any the incubated plates.

The secondary exposure to live bacteria (challenge injections) was carried out 7 days after the previous exposure (Pham et al., 2007; Kutzer et al., 2019). Before the injections, the survival of pre-exposed flies was assessed. For the injections, flies were anesthetised and injected on the left side of the thorax with a volume of 18.4 nL of live bacterial solution or *Drosophila* Ringer’s solution. Therefore, flies injected with *L. lactis* were given a dose of approximately 92 CFUs and those injected with *P. burhodogranariea* were given a dose of approximately 920 CFUs (Acuña Hidalgo et al., 2021). Across experimental repeats, 138 flies per primary exposure treatment were injected with either live *L. lactis* or *P. burhodogranariea* (24–30 flies per repeat), and 78 were injected with Ringer’s solution (12–18 flies per repeat). After the challenge injections, flies were placed in vials containing fresh SYA medium in groups of six flies (Kutzer et al., 2018) and flipped into new food vials every 3–4 days. A single aliquot of bacterial solution or Ringer’s solution was used for each group of six flies and each of them was plated at the end of the injections to check for potential contamination, which we did not find. Additionally, to verify the dose of bacteria that had been injected we prepared three serial dilutions from 1:1 to 1:10^4^ for *L. lactis* and 1:1 to 10^5^ for *P. burhodogranariea*. Eight droplets of 5 μL per dilution were plated for the three highest dilutions before and after the challenge injections, and counted after 20 h of incubation at 30°C. From these counts we estimated that the injected doses were thereby on average 136 ± 5.22 CFUs for *L. lactis* and 1,168 ± 37.60 CFUs for *P. burhodogranariea*.

### Survival and Bacterial Load Assays

For three of the five experimental replicates, a portion of the vials from each replicate were randomly allocated to survival, which was monitored daily for 7 days for a total of 96 bacteria-infected flies per primary exposure and challenge treatment (18–30 flies per experimental repeat), and for a total of 60 Ringer’s injected flies (12–18 flies per experimental repeat). The remaining vials from each replicate were randomly allocated to bacterial load measures. For the two other replicates, we monitored only survival as described above.

For bacterial load measures, at one- and 7-days post-challenge, flies from randomly allocated vials were homogenised. A total of 21 flies per previous exposure and challenge treatment (seven flies for each of the three experimental repeats) were allocated to each timepoint. For homogenisation, flies were anesthetised with CO_2_, removed from their vial, and transferred into a 1.5 mL microcentrifuge tube containing 100 μL of LB media and one stainless steel bead (Ø 3 mm, Retsch) and immediately placed on ice. The tubes were placed in a Retsch Mill (MM300) inside holders that had been previously chilled for 30 min at 4^°^C. The flies were homogenised at a frequency of 20 Hz for 45 s. The tubes were subsequently centrifuged at 420 rcf for 1 min at 4^°^C. The homogenate was re-suspended and 80 μL were placed in a 96-well plate, and one serial dilution from 1:10 to 1:10^5^ was performed for each sample. For each of the six dilutions, three droplets of 5 μL per fly were placed onto LB agar and incubated at 30^°^C for approximately 20 h. The number of CFUs per droplet were counted for the dilutions with droplets containing between approximately 10–60 CFUs. The bacterial load per fly was estimated by averaging the counts for the three droplets and back-calculating the number of CFUs in each fly based on the number of dilutions. *D. melanogaster* microbiota does not easily grow under the above culturing conditions (e.g., Hanson et al., 2019; Kutzer et al., 2019). Nonetheless we homogenised flies that had been challenged with Ringer’s as a control. Of the 145 Ringer’s-injected flies, four flies had more than 2 CFUs in the 1:1 dilution. Of the remaining 439 bacteria-challenged flies, 11 flies (six challenged with *L. lactis* and five challenged with *P. burhodogranariea*) had more than 2 CFUs and were excluded from the analyses. One of the *L. lactis*-injected flies had too many CFUs to count in the highest dilution factor (1:10^5^), therefore, its bacterial load was replaced by the highest bacterial load from the same bacteria, experimental replicate and day post-challenge, i.e., 3,133,333 CFUs.

### Statistical Analyses

All statistical analyses were performed using R studio (R version 3.6.3). Figures were created using plyr (Wickham, 2011), dplyr (Wickham et al., 2020), and ggplot2 (Wickham, 2009). For each model, the effects of the explanatory variables and interactions on the response variable were tested using a Wald test (Bolker et al., 2009). As explanatory variables, all the models included the previous exposure treatment and the experimental repeat, as well as the interaction between these two variables unless stated otherwise. For all the analyses, each model was tested independently for each bacterial species, and the same group of control flies, i.e., injection with Ringer’s was used as the control.

We tested the effect of the previous exposure treatment on survival 7 days after the pre-exposure, by comparing the survival of dead bacteria-injected flies to Ringer’s injected flies. We used a generalised linear models *glm* binomial errors. Using the function cbind, the number of flies that died and the number of flies that survived per vial was combined into a vector, which we used as a response variable. Previous exposure treatment (F: formaldehyde inactivation, HK: heat-killing, F + HK: equal parts of bacterial cells inactivated with heat-killing or formaldehyde), experimental repeat, and their interaction, were used as factors. Model 1a tested the survival after pre-exposure for *L. lactis*, and model 1b for *P. burhodogranariea*:


Models 1a,b:Survivalpost-primingL.lactis,P.b⁢u⁢r⁢h⁢o⁢d⁢o⁢g⁢r⁢a⁢n⁢a⁢r⁢i⁢e⁢a∼Previous⁢exposure×Repeat

The effect of previous exposure on survival post-challenge was tested by comparing the survival of flies infected with either *L. lactis* or *P. burhodogranriea* that had been previously exposed to Ringer’s solution, formaldehyde inactivated bacteria, heat-killed bacteria or the pre-exposure treatment containing both types of inactivated bacteria. We tested for differences in survival using *coxme* in the *survival* package (Therneau and Grambsch, 2000; Therneau, 2020). Previous exposure treatment and experimental repeat, and their interaction, were included as factors. The identification number of the vial that the flies had been kept in for the survival assay was included as a random effect. The variable tested was a survival object constructed for each individual fly with the function *Surv* in the survival package. This vector contained two variables: a binary censor variable that indicates whether the fly was dead (1) or alive (0), and the day at which the fly died, or in the case of censored flies (i.e., that were still alive at the end of the assay) the last survival check day (7 days post-infection). Models 2a and 2b tested survival after a challenge with *L. lactis* and *P. burhodogranariea*, correspondingly:


Models 2a,b:Survivalpost-challengeL.lactis,P.b⁢u⁢r⁢h⁢o⁢d⁢o⁢g⁢r⁢a⁢n⁢a⁢r⁢i⁢e⁢a∼Previous⁢exposure×Repeat+(1|Vial⁢ID)

For both bacterial species, visual inspection of the log10 transformed bacterial load + 1 suggested that the data distribution on day one post-challenge was not unimodal. This was statistically confirmed using a Hartigan’s Dip test for unimodality (Hartigan and Hartigan, 1985) with the *dip.test* function from the *diptest* package (Maechler, 2016) by simulating 5,000 *p*-values (see Results Section). The k-means clustering method (Forgy, 1965; MacQueen, 1967; Hartigan and Wong, 1979; Lloyd, 1982), suggested that the data is bimodal. We therefore sub-set the bacterial load data for day one post-challenge into two groups. We determined the cut-off point between these groups as the local minima in the interval between the highest values for both modes. For *L. lactis* the cut-off value was 18268.63 CFUs and for *P. burhodogranariea* it was 14383.78 CFUs. We divided the data into two subsets comprised of flies with a “low” (i.e., below the cut-off point) or “high” (above the cut-off point) bacterial load. Both subsets were analysed separately for each bacterial species. The effect of the previous exposure treatment and experimental repeat on bacterial load was tested with a linear model on a log10 transformation of the bacterial load + 1 using the *lm* function. Models 3a and 3b were for the low and high subsets of flies infected with *L. lactis*, respectively, and models 3c and 3d for the low and high subsets infected with *P. burhodogranariea*, respectively. Additionally, we detected three data points in model 3c, which could potentially have been influential (i.e., they were either around or above 0.5 Cook’s distance). We therefore additionally analysed the low subset of data without these three data points (model 3e) as a generalised linear model with quasipoisson distribution. Both models 3c and 3e gave qualitatively similar results (see Supplementary Table 2 for the results from model 3e). We did not include the identity of the vial in which flies had been kept as a random variable, because the flies were sampled at random from the vials. We did not include the interaction between previous exposure and repeat because some combinations of previous exposure and experimental repeat contained only one individual.


Models 3a,b,c,d:Log10(Bacterialloadday 1L.lactisorP.burhodogranariea+1)∼Previousexposure+Repeat

Models 3e:Bacterialloadday 1P.burhodogranariea∼Previous⁢exposure+Repeat

Given the bimodal distributions described above, we reasoned that in addition to the bacterial load *per se*, the primary exposure could also affect the proportion of flies in the high and low groups. Therefore, for each replicate, using the function cbind we created a response vector, y, containing the number of flies in the low group, and the number of flies in the low group subtracted from the total number of flies in the low and the high group. This was entered into a *glm* with binomial error for the *L. lactis* load. We used a quasibinomial error for the *P. burhodogranariea* load to account for over dispersion of the data. Previous exposure treatment was given as the factor.


Models 4a,b:yL.lactisorP.burhodogranariea∼Previous⁢exposure

Bacterial load data 7-days post-challenge was found not to differ significantly from a unimodal distribution using the Hartigan’s Dip test for unimodality as described above. The effect of previous exposure treatment on the bacterial load 7 days post-challenge was tested using a linear model with log10 transformed bacterial load + 1 (Model 4a for a challenge with *L. lactis*, and 4b for *P. burhodogranariea*).


Models 5a,b:Log(Bacterialloadday 7L.lactisor P.burhodogranariea+1)∼Previousexposure×Repeat

## Results

### Survival After a Previous Exposure to Inactivated Bacteria

Fly survival directly before the live bacterial challenge was higher than 96% across all treatments and experimental repeats (Supplementary Figure 1). There was no significant effect of the previous exposure treatment or experimental repeat for either bacterial species, and there was no interaction between these two factors (Supplementary Table 1).

### Survival After a Live Bacterial Challenge

**FIGURE 2:**
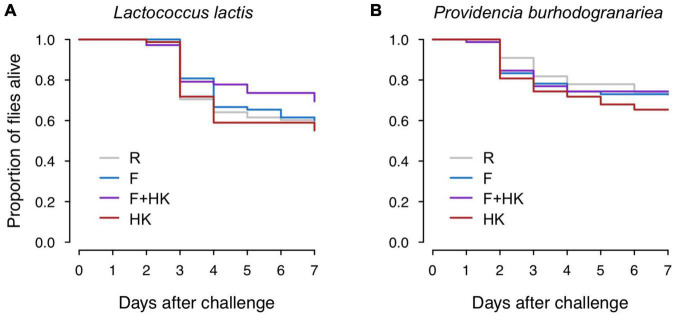
Effect of the previous exposure on survival 7 days post-challenge. Flies were challenged with either **(A)** 92 colony forming units (CFUs) of *Lactococcus lactis*, or **(B)** 920 of *Providencia burhodogranariea*. Previous exposure treatments are *Drosophila* Ringer’s solution (R), formaldehyde-inactivated bacteria (F), a mixture of formaldehyde-inactivated and heat-killed bacteria (F + HK), and heat-killed bacteria (HK). Survival did not differ significantly according to previous exposure treatment. For statistics, see Table 1.

As expected, fly survival was high 7 days after challenge with *Drosophila* Ringer’s, and it was unaffected by the previous exposure injection: across all seven control groups there was 98.95% survival (three out of 287 flies died). Contrary to our expectations, we did not find any significant differences in survival between flies injected with the different pre-exposure treatments, whether they were challenged with *L. lactis* or *P. burhodogranariea* (Table 1 and Figure 2), meaning that there were no survival benefits to any of the primary bacterial exposure treatments compared to the Ringer’s primary control exposure.

**TABLE 1:** The effects of previous exposure and experimental repeat on fly survival for the 7 days post-challenge.

	Model 2a: *L. lactis*			Model 2b: *P. burhodogranariea*		
Tested effect	Chi square	df	*P*	Chi square	df	*P*
Previous exposure	3.22	3	0.36	2.22	3	0.53
Repeat	0.98	2	0.61	2.71	2	0.26
Previous exposure × repeat	7.18	6	0.30	11.81	6	0.066

*Previous exposure treatments include Drosophila Ringer’s solution, or bacteria that had been inactivated in different ways, i.e., formaldehyde-inactivated bacteria, heat-killed bacteria or a mixture of the two. Flies were then injected with a homologous live challenge of either L. lactis (Model 2a) or P. burhodogranariea (Model 2b).*

### Resistance After a Live Bacterial Challenge

**FIGURE 3:**
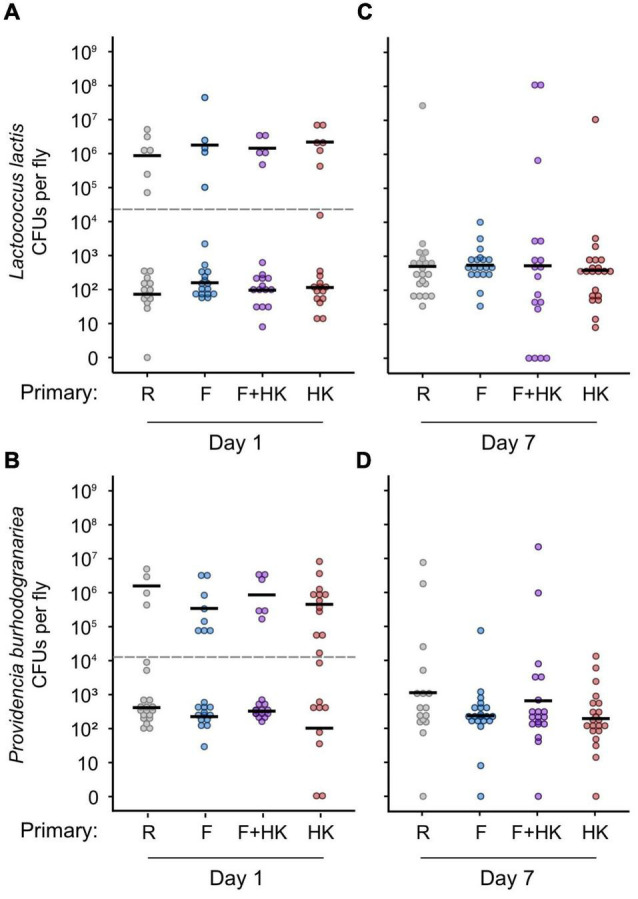
Bacterial load of individual flies 1 and 7 days after a homologous challenge with **(A,C)** 92 colony forming units (CFUs) of *Lactococcus lactis*, or **(B,D)** 920 CFUs of *Providencia burhodogranariea*. Bacterial load on the y-axis was quantified as the number of colony-forming units per fly. Here, we present a log transformation of the CFU (+1) for ease of interpretation. On the x-axis, previous exposure treatments are presented as *Drosophila* Ringer’s solution (R), formaldehyde-inactivated bacteria (F), a mixture of formaldehyde-inactivated and heat-killed bacteria (F+HK), and heat-killed bacteria (HK). Bacterial load at day one is in the left-hand column, and the load at day seven is in the right-hand column. Black lines show the geometric mean of the bacterial load per treatment, and per subset for bacterial load 1-day post-challenge. The grey dotted lines represent the cut-off points dividing the low and high bacterial load subsets, which were analysed separately. We did not find any effect of the previous exposure on bacterial load for either of the 2 days assayed. For statistics, see Table 2.

Host resistance, i.e., the inverse of bacterial load was assessed on days one and seven after challenge (Figure 3). We found that eleven flies across both days cleared the infection: five out of 157 flies had no *L. lactis* CFUs, and six out of 156 flies had no *P. burhodogranariea* CFUs.

On day one post-challenge, regardless of treatment and bacterial species, bacteria-infected flies showed large variation in their bacterial load (Figures 3A,B). The data did not follow a unimodal distribution (*L. lactis: D* = 0.071, *p* = 0.0026; *P. burhodogranariea: D* = 0.072, *p* = 0.0026), with some flies showing a high bacterial load while most flies had a lower bacterial load. Therefore, by calculating the local minima between the highest values for each group of flies, a cut-off point was determined to split the data into two subsets. The data were analysed separately for flies belonging to the low (below the cut-off point) or high (above the cut-off point) subsets (Figures 3A,B) for both bacterial species. We found for both subsets and bacterial species that the pre-exposure treatment did not have a significant effect on the mean bacterial load on day one post-challenge (Table 2). For *P. burhodogranariea*, experimental repeat had a significant effect on the bacterial load of the low subset (Table 2). This effect was mainly driven by the presence of a replicate with two flies pre-exposed with heat-killed bacteria that cleared the infection, as clearance was not found in any other treatment for this bacterial species and day post-challenge. We found that previous exposure treatment did not affect the proportion of flies in the high and the low load groups for either flies infected with *L. lactis* (Chi square = 0.37, df = 3, *p* = 0.95) or *P. burhodogranariea* (Chi square = 4.00, df = 3, *p* = 0.26).

**TABLE 2:** The effects of previous exposure and experimental repeat on bacterial load on day one post-challenge.

*L. lactis*	Model 3a: low subset			Model 3b: high subset		
Tested effect	*F*	df	*p*	*F*	df	*p*
Previous exposure	0.89	3,51	0.45	0.40	3,16	0.75
Repeat	1.62	2,51	0.21	0.82	2,16	0.46

***P. burhodogranariea***	**Model 3c: low subset**			**Model 3d: high subset**		
**Tested effect**	***F***	**df**	***p***	***F***	**df**	***p***

Previous exposure	2.36	3,45	0.084	1.02	3,24	0.40
Repeat	7.19	2,45	**0.002**	1.32	2,24	0.29

*Bacterial load data was split into “low” and “high” subsets by cutting off the data at the local minima between the highest bacterial load values for each subset. These subsets were analysed separately. Previous exposure treatments include Drosophila Ringer’s solution, formaldehyde-inactivated bacteria, heat-killed bacteria or a mixture of the two. Flies were then injected with a homologous live challenge of 92 colony forming units (CFUs) of L. lactis (Models 3a and 3b), or b: 920 of P. burhodogranariea (Models 3c and 3d). Statistically significant factors are shown in bold.*

On day seven post-challenge, the bacterial load for the two bacterial species did not differ significantly from a unimodal distribution (*L. lactis*: *D* = 0.040, *p* = 0.48; *P. burhodogranariea*: *D* = 0.026, *p* = 0.99) (Figures 3C,D). We did not find any significant effect of the priming treatment on the bacterial load 7 days after challenge (Table 3).

**TABLE 3:** The effects of previous exposure and experimental repeat on bacterial load on day seven post-challenge.

	Model 5a: *L. lactis*			Model 5b: *P. burhodogranariea*		
Tested effect	*F*	df	*P*	*F*	df	*P*
Previous exposure	0.043	3	0.99	1.39	3	0.25
Repeat	2.27	2	0.11	1.15	2	0.32
Previous exposure × repeat	0.25	6	0.96	0.39	6	0.88

*Previous exposure treatments include Drosophila Ringer’s solution, formaldehyde-inactivated bacteria, heat-killed bacteria, or a mixture of the two. Flies were then injected with a homologous live challenge of 92 colony forming units (CFUs) of L. lactis (Model 5a), or b: 920 of P. burhodogranariea (Model 5b).*

## Discussion

Our study addresses whether pre-exposure to two bacterial species inactivated with different methods, affects subsequent host survival and resistance against a secondary challenge. We found no enhanced host survival or resistance after a primary exposure to dead bacteria, which was consistent across inactivation treatments and bacterial species. Our results highlight the dynamic nature of host resistance over the infection course, and they raise questions as to whether immune priming is a universal trait of insect immunity.

### Pre-exposure Treatment Does Not Affect Survival

As predicted, we found that a primary injection with inactivated bacteria resulted in high survival (>96%) and similar mortality compared to a primary injection with Ringer’s solution. We used dead bacteria for the primary exposure, which for priming experiments has potential advantages over live bacteria: first there is usually minimal mortality after injection with dead bacteria meaning that unlike after the injection of live bacteria, there is no self-selection for a sub-group of fitter flies that survive until challenge; in the case of live bacterial injection, these latter flies may themselves then be predicted to have increased survival after a second infection. Second, a primary exposure with live bacteria will likely reach varying densities across flies by the time of the secondary challenge or even be cleared (Duneau et al., 2017; Acuña Hidalgo et al., 2021); this will result in heterogeneity in the immunological history of the population of flies that are to be challenged. Bacterial infections in insects have been shown to be highly persistent and to lead to sustained antimicrobial responses in the host (Haine et al., 2008; Chambers et al., 2019; Acuña Hidalgo et al., 2021). Using live bacteria for the pre-exposure can lead to persistent infections inside the host, as well as the maintenance of a high level of immune activity, in turn advantaging the host when fighting a secondary bacterial infection (Chambers et al., 2019). However, it is important to note that immune priming responses to inactivated bacteria can persist over time, e.g., antimicrobial responses to heat-killed *S. aureus* can be sustained in *T. molitor* for at least 21 days (Makarova et al., 2016).

### Pre-exposed Flies Have Neither Increased Survival nor Resistance, but Resistance Varies Over the Course of Infection

An advantage of a pre-exposure to fighting a secondary bacterial challenge has most frequently been measured in terms of increased survival to the secondary infection (Boman et al., 1972; Faulhaber and Karp, 1992; Pham et al., 2007; Roth et al., 2009; Christofi and Apidianakis, 2013; Lin et al., 2013; Miyashita et al., 2014; Wu et al., 2014; Futo et al., 2016; Castro-Vargas et al., 2017; Lafont et al., 2017; Dhinaut et al., 2018). Contrary to our expectations, we did not find that pre-exposed flies survived the bacterial challenge better than non-exposed flies. Although less commonly tested in the context of immune priming, host resistance, as measured by pathogen load, has been shown to be increased in hosts previously exposed to pathogens (Boman et al., 1972; Sadd and Schmid-Hempel, 2006; Pham et al., 2007; Miyashita et al., 2014). However, we did not find pre-exposed hosts to be more resistant to a live bacterial challenge in the acute (1 day post-challenge) or chronic (day seven post-challenge) phases of infection. While our results contrast with some pathogen infections in *D. melanogaster* (Boman et al., 1972; Pham et al., 2007), they are consistent with those of a recent study by Kutzer et al. (2019) which showed that four inbred fly genotypes pre-exposed to heat-killed *L. lactis* did not have a higher survival in the 28 days post-homologous challenge, and they did not have increased resistance one and 28 days post-challenge (Kutzer et al., 2019). Despite using a lower challenge dose in our current study, an outbred fly population, and different antigen production methods, the results of the two studies are consistent in that pre-exposure does not offer any significant advantages.

While resistance did not differ between pre-exposure treatments, bacterial load varied over the course of the challenge infection. One day post-challenge, bacterial load followed a bimodal distribution, consistent with previous data on the dynamics of bacterial infections (Duneau et al., 2017). Duneau et al. (2017) showed that the early dynamics of bacterial load follow a bimodal distribution for intermediately virulent bacterial species, with different predicted outcomes of infection for each of the modes. Hosts with high pathogen burden are not able to control the infection and will die during the acute phase of infection. Meanwhile, other hosts will manage to control the pathogen growth and will survive, entering a phase of chronic infection with a constant pathogen load, the set point bacterial load (Duneau et al., 2017). We expected that, if the primary exposure affected acute phase resistance, it would be apparent in the proportion of flies in the high versus low sub-group, or it would be apparent in the resistance of the flies in the lower subgroup. However, primary exposure affected neither of these responses. Seven days after infection, we observed that clearance of the bacteria was rare, and bacterial load was unimodally distributed. Our results highlight the importance of measuring bacterial load as a measure of resistance at more than one point during the infection.

### Resistance Is Not Influenced by the Inactivation Method

Heat-killing (Pham et al., 2007; González-Tokman et al., 2010; Longdon et al., 2013; Wu et al., 2014; Riessberger-Gallé et al., 2015; Kutzer et al., 2019) and formaldehyde-inactivation (Wang et al., 2009; Zhuang et al., 2011; Dhinaut et al., 2018) are two of the most frequently used methods to inactivate pathogens in priming studies. Based on Lin et al. (2013) we had reason to hypothesise that host responses would vary according to the inactivation protocol and to our knowledge, a combination of these two methods has not been tested before. Based on the properties of both types of antigenic preparations, we predicted that combining bacterial cells inactivated with both treatments would result in a synergistic effect in which hosts would benefit from the high antigenicity of formaldehyde-inactivated bacteria, and a fast trigger of the immune response caused by the lipopolysaccharides freed upon cell membrane disruption during heat-killing (Lin et al., 2013). However, our results showed that the method used to inactivate the bacteria for the pre-exposure did not influence host resistance. It could be that these treatments still induce differential immune responses in terms of strength, speed and duration (Pradeu and Du Pasquier, 2018) but lead to similar outcomes in terms of bacterial load, however we did not test this. Interestingly host survival in the Lin et al. (2013), study was not different between hosts pre-exposed to different antigen preparations despite the differences measured in the immune response to both types of inactivated bacteria (Lin et al., 2013).

### Can We Consider Priming as a Ubiquitous Aspect of Innate Insect Immunity?

Our study offers a multi-angled evaluation of the effects of pre-exposure on a secondary challenge. Despite this, we did not find any advantage of previous exposure against a bacterial infection across any pre-exposure treatments. Other studies have identified a priming response in *D. melanogaster* (Boman et al., 1972; Pham et al., 2007) but similar to our study, priming is not always found (Pham et al., 2007; Reber and Chapuisat, 2012; Kutzer et al., 2019). In addition, many experimental parameters can be explored to achieve priming, including the pre-exposure and challenge doses and bacterial species. While *L. lactis* and *P. burhodogranariea* were isolated from *D. melanogaster* (Lazzaro, 2002; Juneja and Lazzaro, 2009), and can cause intermediate virulence and persistent infections (Acuña-Hidalgo, Silva & Armitage, personal observation), it could be that pre-exposure against other pathogens with different infection dynamics might result in other outcomes. For example, Kutzer et al. (2019) found that pre-exposure with heat-killed *Pseudomonas entomophila*, a more virulent bacterium than the two bacteria tested in this study resulted in a lower resistance across genotypes (Kutzer et al., 2019). A theoretical consideration of immune priming suggested that virulence plays a role in how a pre-exposed host will respond to the infection (Best et al., 2013). Tolerance is another host defence strategy that quantifies the ability of the host to maintain its fitness in the face of an infection (Råberg et al., 2009), and which has been rarely explored in priming studies (but see Kutzer et al., 2019). In the case of fecundity as a measure for fitness, Kutzer et al. (2019) found no effect of previous exposure on fecundity-tolerance, and although we did not explicitly test it here, the fact that survival and bacterial load did not differ across treatments suggests no effect of survival tolerance under these experimental conditions.

Finally, as mentioned above, while our study and several others did not find support for priming, it might be that this phenomenon only occurs only under certain circumstances, such as specific host-pathogen combinations (Roth et al., 2009; Pope et al., 2011). For instance, Pope et al. (2011) found that white shrimp can be primed using the bacteria *Vibrio harveyi* but not *Bacillus subtilis.* They argued that shrimp pre-exposed to *V. harveyi* might have an advantage against a live challenge since this bacterium is a known pathogen present in the host natural environment, to which the host may have evolved priming defences, while *B. subtilis* is not naturally present in this environment (Pope et al., 2011). Because it allows the host to reduce or avoid the negative effects of an infection on host fitness, immune priming might be expected to be subjected to a strong selection pressure (Best et al., 2013). However, if it is the case that priming is only elicited in specific experimental circumstances, one could argue about the adaptive value of this phenomenon. Immune priming might then not be a general trait of the innate immune system, but rather a defence trait specific to populations where it gives a significant evolutionary advantage against pathogens.

## Data Availability Statement

The data presented in this study are publicly available: http://dx.doi.org/10.17169/refubium-33781.

## Author Contributions

SA conceived the idea and designed the experiments together with BA. BA collected the data, conducted the statistical analyses with advice from SA, and wrote the first draft of the manuscript. Both authors contributed critically to the drafts and gave final approval for publication.

## Conflict of Interest

The authors declare that the research was conducted in the absence of any commercial or financial relationships that could be construed as a potential conflict of interest.

## Publisher’s Note

All claims expressed in this article are solely those of the authors and do not necessarily represent those of their affiliated organizations, or those of the publisher, the editors and the reviewers. Any product that may be evaluated in this article, or claim that may be made by its manufacturer, is not guaranteed or endorsed by the publisher.
