# Supplementary material for: Host Resistance to Bacterial Infection Varies Over Time, but Is Not Affected by a Previous Exposure to the Same Pathogen
Source: Front Physiol. 2022 Mar 21;13:860875. doi: 10.3389/fphys.2022.860875 (PMC8979062; doi:10.3389/fphys.2022.860875)
Supplement: Supplementary file 1 [file Data_Sheet_1.docx]

Supplementary Material

# Supplementary Figure and Tables

## Supplementary Figure

**Supplementary Figure 1.** Proportion of flies that were alive before challenge, i.e., seven days after the initial exposure for flies previously exposed to (**A**): *L. lactis*, or (**B**): *P. burhodogranariea*. Flies were previously exposed to one of the following treatments: *Drosophila* Ringer’s solution (R), formaldehyde-inactivated bacteria (F), a mixture of formaldehyde-inactivated and heat-killed bacteria (F+HK), heat-killed bacteria (HK). Mean survival and standard error are shown for all each pre-exposure treatment. For statistics, see Table S1.

## Supplementary Tables

Table S1. The effects of previous exposure treatment and experimental repeat on survival seven days after the previous exposure, i.e., immediately before the challenge. Flies were previously exposed to either *Drosophila* Ringer's solution, bacteria from the species *L. lactis* (Model 1a) or *P. burhodogranariea* (Model 1b) that were formaldehyde-inactivated or heat-killed bacteria, or a mixture of bacteria inactivated with these methods.

|  | Model 1a: *L. lactis* | | |  | Model 1b: *P. burhodogranariea* | | |
| --- | --- | --- | --- | --- | --- | --- | --- |
| Tested effect | Chi square | df | P |  | Chi square | df | p |
| Previous exposure | 1.87 | 3 | 0.76 |  | 0.34 | 3 | 0.95 |
| Repeat | 2.94 | 4 | 0.71 |  | 5.33 | 4 | 0.25 |
| Previous exposure × repeat | 2.14 | 12 | 1.00 |  | 5.65 | 12 | 0.93 |

**Table S2.** The effects of previous exposure and experimental repeat on bacterial load of the low subset on day one post-challenge with live *P. burhodogranariea*, when three potentially influential data points were removed from the analysis. Bacterial load data was split into “low” and “high” subsets by cutting off the data at the local minima between the highest bacterial load values for each subset. Previous exposure treatments include *Drosophila* Ringer's solution, formaldehyde-inactivated bacteria, heat-killed bacteria or a mixture of the two. Statistically significant factors are shown in bold.

|  | Model 3e: low subset | | |
| --- | --- | --- | --- |
| **Tested effect** | **Chi square** | **df** | **p** |
| Previous exposure | 6.17 | 3 | 0.10 |
| Repeat | 35.77 | 2 | **< 0.001** |
